# Envisioning Better Healthcare Systems: A Unique and Innovative Journey From Community Hospital to Academic Medical Center

**DOI:** 10.7759/cureus.41032

**Published:** 2023-06-27

**Authors:** Nancy Finnigan, Andrew R Barbera, Donald Davis, Luis D Lugo, Sean McCann, Shirley Alleyne, Melissa Rusli, Walter Wills, Andrew Bugajski

**Affiliations:** 1 Medical Education, Lakeland Regional Health, Lakeland, USA; 2 Research and Sponsored Studies, Lakeland Regional Health, Lakeland, USA

**Keywords:** academic residency, transition to residency curriculum, accreditation, medical residency, global healthcare system

## Abstract

The transformation from a community hospital to an academic medical center (AMC) presents a unique set of challenges and opportunities. This editorial provides an in-depth analysis of the barriers encountered and solutions developed within a large community hospital in Florida as it embarked on this transition, with a focus on the global relevance of issues experienced such as competition with major markets, the ongoing COVID-19 pandemic, the development of multiple Accreditation Council for Graduate Medical Education (ACGME) programs and balancing the complexities of the United States healthcare system. In alignment with the call for submissions, this editorial highlights the personal experiences of healthcare providers, researchers, and policymakers involved in this transition and explores how the lessons learned can inform the development of better healthcare systems worldwide.

## Editorial

Healthcare systems across the globe face significant challenges including issues related to quality, cost, equity, innovation, and regulation [1-3]. However, successful healthcare models can emerge from surprising locations, offering valuable insights into how we can address these challenges and improve patient care and outcomes. The transition of a large community hospital in Florida, United States of America to an academic medical center (AMC) provides an opportunity to examine the unique and innovative approaches taken to navigate the complexities of the United States healthcare system while addressing global health issues.

Competing in the global academic medical landscape

In an increasingly interconnected world, academic medicine has become a global enterprise; institutions must compete for resources, talent, and prestige on a worldwide scale [4]. Physicians and researchers at this community hospital have leveraged their personal experiences to create a unique value proposition, emphasizing the institution's strengths and differentiating it from other AMCs. By fostering international collaborations, participating in global health initiatives, and integrating diverse perspectives from healthcare providers, policymakers, and patients, the hospital can enhance its global reputation and contribute to the development of better healthcare systems worldwide.

The key to being an effective competitor begins with the leadership team [5]. During the initial transition period of the institution to an AMC, executive leadership within the institution focused recruiting efforts related to the medical education program on the recruitment of academic, self-motivated, insightful, and innovative leaders to cultivate a framework for a strong academic culture. By recruiting leaders with international and local connections, the leadership team cultivated a diverse perspective of voices when considering the development of the institution's academic programs.

In developing the specific curriculum and strategic plan of the institutional residency programs, each specialty program director reviewed the current landscape of residency programs associated with AMC's nationally and locally to aid in their own program's development, focusing on what characteristics distinguished those programs across the United States as successful student experiences. Special attention was given to research initiatives, simulation offerings, and wellness programs [6,7]. The team also engaged with various community voices, generating excitement for the residents’ arrival and explaining the benefits of becoming an AMC to the community at large. By engaging the marketing department, the team was able to share our plan in multiple media formats (via program websites and social media marketing). Keeping in mind the National Resident Matching Program statistics regarding resident ranking preferences, our team placed high emphasis on ensuring the residents had a memorable interview day experience.

Internally, the daunting task of obtaining strategic buy-in from the institution’s stakeholders and faculty began with the conception of the programs. Faculty development initiatives were the mainstay of faculty engagement, including retreat offerings and early feedback mechanisms regarding faculty engagement prior to the arrival of residents. As with many new residency programs and illustrated by Becker et al., faculty scholarly activity was an area of opportunity [8]. By creating a business case for the need for research to support the residency programs, the team was able to create an onsite research department with dedicated resources for assisting residents and hosts an interdisciplinary regional research symposium onsite.

As experienced during the initial transitions of other AMCs [9], the analysis of the significant economic impact to the community brought by a large graduate medical education program was shared with elected officials in an effort to secure additional sources of grant funding for start-up costs to offset the costs of such a large investment. Multiple resources are available to assist with the analysis, including data from the Association of American Medical Colleges.

Addressing the impact of the COVID-19 pandemic

The COVID-19 pandemic has highlighted both the shortcomings and successes of healthcare systems around the world (i.e., shifting trends in healthcare utilization [10], the spread of vaccine misinformation [11], and global dissemination of rapid screening tools [12]). The hospital's response to the pandemic demonstrates the importance of adaptability and innovation in the face of unprecedented challenges. As one of the busiest emergency departments in the world [13], adaptability is one of the core values of the healthcare professionals who work there. Rapid engagement with emergency guidelines and the evolution of the system’s profile set a groundwork of innovation and disruption that is often not seen in healthcare. Physicians, public health researchers, and policymakers have collaborated to implement novel approaches to patient care and medical education, such as telemedicine and online learning. By sharing their experiences, these healthcare professionals can contribute to a global dialogue on effective pandemic response strategies and inform the development of more resilient healthcare systems.

As demonstrated by Hogan et al. and evidenced by the experience of this transitioning hospital system [14], the COVID-19 pandemic had significant impacts on graduate medical education, particularly related to the delay in ACGME site visits, which further delayed accreditation, the start and timing of the residency programs, and increased difficulty in recruitment related to travel restrictions [14,15]. Additionally, the supply chain challenges resulted in delayed construction projects for the resident lounge and call quarters, subsequently delaying the start of the programs.

Building an ACGME-accredited program with global relevance

Developing an ACGME-accredited program requires significant investment in resources, infrastructure, and personnel [16]. The Florida hospital's journey highlights the dedication of healthcare providers and policymakers to create a program that not only meets the rigorous standards of accreditation but also addresses global health issues. By incorporating global health competencies addressed by Welten et al. [17], promoting international collaborations, and providing opportunities for trainees to engage in cross-cultural learning experiences, the hospital's program prepares residents for practice in an increasingly interconnected world, equipping them with the skills necessary to address global health challenges.

Balancing within the United States healthcare system and beyond

Navigating the complexities of the United States healthcare system presents unique challenges [18]. As Rice et al. eloquently identifies, the United States excels in the quality and training of its clinicians and researchers, however, is simultaneously saddled with significant inequities in healthcare coverage and demonstrates excessive healthcare service expenditures [18]. Nonetheless, the experiences of physicians, public health researchers, and policymakers at this hospital demonstrate the potential for innovation and collaboration in the face of these challenges. By partnering with local community organizations and public health agencies, the hospital has successfully addressed disparities in access to care and created a model that prioritizes equity and social responsibility. Furthermore, the institution's diversified funding strategy ensures the long-term sustainability of its academic programs while minimizing the impact on healthcare costs for patients.

With one of the busiest emergency departments in the country [13], our health system serves a large portion of underserved patients with barriers to accession to care. Our residency clinics provide an avenue to patients that are using the emergency department for primary care due to a lack of insurance or underinsurance. By implementing an all-payor approach to our clinics and providing appropriate social services support in those clinics, we are able to begin to address the barriers to accession of care within a vulnerable patient population.

The transition from a community hospital to an AMC in the United States offers valuable insights into the development of better healthcare systems worldwide. By sharing the personal experiences of healthcare providers, researchers, and policymakers involved in this transition, we can foster a global dialogue on important issues that inevitably impact patient care and outcomes. This institution's journey serves as an inspiring example for other community hospitals contemplating a similar transition, demonstrating the potential for innovation, collaboration, and excellence in the face of significant challenges.

As we envision better healthcare systems for all, it is crucial to learn from successful models like this Florida community hospital's transition to an AMC. By examining the unique approaches taken in addressing competition, the COVID-19 pandemic, the development of an ACGME-accredited program, and the complexities of the United States healthcare system, we can derive valuable lessons that can inform healthcare systems worldwide. By sharing these perspectives and engaging in a global dialogue, we can work together to create more equitable, accessible, and effective healthcare systems that improve patient care and outcomes for all.

As we move forward, it is essential to continue monitoring and evaluating the progress of this Florida community hospital's transition to an AMC. Identifying both the successes and areas for improvement will enable other institutions to learn from this experience, replicate the best practices, and avoid potential pitfalls. In addition, fostering ongoing collaboration and communication among healthcare providers, researchers, and policymakers will help address emerging challenges and opportunities in the rapidly evolving healthcare landscape.

Future research should explore the long-term outcomes of the hospital's transition, including the impact on patient care, satisfaction, and health outcomes, as well as the experiences of medical residents and faculty. Additionally, comparative studies of similar transitions in other institutions and countries can provide further insights into best practices and potential barriers to success.

Ultimately, the journey of this Florida community hospital serves as a powerful reminder of the potential for innovation and collaboration in the face of seemingly insurmountable challenges. By learning from this experience and working together, we can strive to create better healthcare systems that serve the needs of all patients, both locally and globally.
